# Evaluation of a large language model for clinician-facing preoperative cost-communication preparation in total knee arthroplasty

**DOI:** 10.3389/fmed.2026.1899060

**Published:** 2026-08-12

**Authors:** Zebing Ma, Liping Xue, Gonghui Jian, Liang Kong, Huayu Fan, Chaofan Yang, Xiaolong Wu, Rui Chen, Xiangyang Cao

**Affiliations:** 1 Hunan University of Chinese Medicine, Changsha, Hunan, China; 2 The First Affiliated Hospital of Hunan University of Chinese Medicine, Changsha, Hunan, China; 3 Luoyang Orthopedic Hospital of Henan Province (Orthopedic Hospital of Henan Province), Zhengzhou, Henan, China; 4 Institute of Intelligent Medical and Bioengineering Henan Academy of Traditional Chinese Medicine Sciences, Zhengzhou, Henan, China; 5 Henan Province Artificial Intelligence Engineering Research Center for Bone Injury Rehabilitation, Zhengzhou, Henan, China; 6 Henan University of Chinese Medicine, Zhengzhou, Henan, China

**Keywords:** clinical text processing, expert-referenced offline evaluation, large language model, preoperative cost communication, total knee arthroplasty

## Abstract

**Background and aims:**

Costs associated with total knee arthroplasty (TKA) may affect treatment preparation, expectation management, and postoperative care planning. Previous large language model (LLM) studies have focused mainly on medical question answering, patient education, and clinical decision support, whereas their performance in clinician-facing preoperative cost-communication preparation remains unclear. This study evaluated an LLM using real-world clinical records from two hospitals in an expert-referenced offline evaluation.

**Methods:**

Preoperative medical records of 80 patients who underwent primary unilateral TKA at two hospitals in China from January to May 2026 were included. A structured expert-panel process was used to develop a preoperative cost-communication framework comprising 4 dimensions and 17 clinical cues and to establish case-level minimum necessary communication items (CL-MNCIs) for each case. Task 1 assessed identification of the 17 cues. Task 2 assessed CL-MNCI coverage and classified all generated items according to case relevance, medical-record support, redundancy, and safety. Twenty-four cases were non-randomly selected by CL-MNCI count for three repeated-generation runs.

**Results:**

Task 1 comprised 1,360 case–label classification units. Micro-precision, micro-recall, micro-F1, accuracy, MCC, and macro-F1 were 0.901, 0.888, 0.894, 0.948, 0.860, and 0.840, respectively. Experts established 569 CL-MNCIs, of which 483 were covered, yielding an overall coverage rate of 84.9%; complete coverage was achieved in 17 cases. The LLM generated 716 items, including 12 safety events across 9 cases, 483 items matching CL-MNCIs, 132 record-supported supplementary items, 50 redundant items, and 39 items with insufficient record support. Overall, 665 items (92.9%) were case-relevant and record-supported, although this proportion included redundant content. Pairwise Jaccard similarity for covered CL-MNCI sets ranged from 0.813 to 0.823. Of 180 CL-MNCIs, 126 (70.0%) were covered in all three runs, and case-level agreement in safety classification ranged from 87.5 to 95.8%.

**Conclusion:**

The LLM showed offline potential for identifying cost-communication cues and generating clinician-facing preparation checklists for TKA, but content omissions, quality variation, safety risks, and substantive cross-run variability remained. Its use should be limited to clinician-reviewed communication preparation and should not replace direct patient cost disclosure or professional judgment.

## 1 Introduction

Total knee arthroplasty (TKA) is an important treatment for end stage knee osteoarthritis (KOA) and can substantially relieve pain and improve joint function (1). However, TKA related medical costs may influence whether patients undergo surgery, when they choose to undergo surgery, and how they weigh persistent pain, functional limitation, and surgical expenses (2). Previous studies on access to knee arthroplasty have shown that affordability, insurance coverage, and socioeconomic status may affect both the opportunity and timing of receiving joint replacement treatment (3). Beyond the financial burden itself, the extent to which cost related information can be translated into clear and discussable preoperative communication content may also influence patients' treatment choices. Evidence from shared decision-making research suggests that direct and indirect care costs can affect patients' health related choices and their ability to act on these choices (4). Therefore, insufficient communication about cost uncertainty, potential additional expenses, and postoperative rehabilitation and care arrangements may increase uncertainty in expectation management, treatment preparation, and postoperative care planning.

Evidence from different health care financing systems suggests that the impact of TKA related costs and payment arrangements on patient decision-making and perioperative care may vary by context. In regions with universal health insurance coverage, the decision of patients with severe KOA to undergo TKA is mainly driven by the impact of joint symptoms on function and quality of life, whereas household income and financial factors have not shown a significant effect (5). In contrast, in multi-payer healthcare systems, insurance payer type is associated with complications after joint arthroplasty, suggesting that the payment context may reflect differences in financial responsibility, baseline patient risk, access to healthcare services, and the need for postoperative support (6). In resource-limited settings, implant costs and patient out-of-pocket expenses may constitute major sources of financial pressure (7). These international findings indicate that preoperative cost communication for TKA is highly context-dependent. In the Chinese healthcare context, differences in medical insurance type, reimbursement scope, regional and institutional processes (8), as well as perioperative management, rehabilitation care, and potential out-of-pocket items (9), may all influence patients' perceptions of cost-related risks and their preparation for treatment. Therefore, preoperative cost communication (POCC) for TKA should not be limited to explaining fixed charges or reimbursement policies. Instead, it should integrate patient-specific clinical risks, degree of functional limitation, surgical complexity, rehabilitation needs, and institutional processes within the specific healthcare setting, thereby helping patients prepare for treatment with a clearer understanding of cost uncertainty.

Large language models (LLMs), represented by ChatGPT, are rapidly evolving, and their potential applications in medical knowledge question answering, clinical text processing, and extraction of information from medical records have attracted broad attention. Previous studies have begun to explore the integration of LLMs into clinical communication workflows. Examples include the integration of LLMs with electronic health records to draft patient-message responses for physicians (10), and their use in supporting patient portal messaging and asynchronous patient–clinician communication (11). In orthopedics, evaluations of LLMs have also increased, including comparisons of clinical knowledge generation by different LLMs in septic arthritis scenarios (12), readability assessments of patient education materials for ankle disorders (13), and assessments of the concordance of LLM-generated recommendations for clavicle fracture management with guidelines from the American Academy of Orthopaedic Surgeons (14). Studies focusing on KOA have mainly examined the accuracy of medical information, the quality of diagnostic and treatment recommendations, or support for clinical decision-making (15). However, POCC for TKA is not simply a task of medical question answering, patient education, or treatment recommendation generation. Rather, it requires clinicians to translate information from preoperative medical records, including comorbidities, functional status, surgical complexity, rehabilitation and care needs, and potential cost uncertainty, into communication points that can be reviewed and discussed. Accordingly, unlike previous LLM studies, this study positioned the LLM as a clinician-facing communication preparation aid. Using real-world preoperative TKA records from two hospitals, we conducted an expert-referenced offline evaluation of the LLM's performance in identifying cost communication-related clinical cues and generating case-level cost communication checklists. We further assessed the coverage, case relevance, medical-record support, safety, and substantive output stability of the generated content.

## 2 Methods

### 2.1 Data sources and case selection

The cases used in this study were sourced from hospitalized patients who underwent TKA between January 2026 and May 2026 at two hospitals in Hunan and Henan Provinces, China. All participants signed informed consent forms, and the study protocol was approved by the ethics committees of each hospital (Approval No: HN-LL-YJSLW-2026-04; 2026Y1SKT0027-03). Inclusion criteria were: (1) age ≥50 years; (2) undergoing primary unilateral TKA for KOA (Kellgren Lawrence grade III/IV); (3) complete preoperative medical record data. Exclusion criteria were: (1) patients who underwent knee arthroplasty for non-degenerative diseases as the main surgical reason, such as emergency fracture, bone tumor, or active infection; (2) patients who underwent other major surgeries during the same hospitalization; (3) patients who underwent revision TKA; (4) patients with missing key preoperative medical records.

Preoperative medical records referred to relevant clinical texts generated before TKA during the same hospitalization, including medical records of patients whose initial department was not orthopedics but who were later transferred to orthopedics because of the need for surgical treatment. Case screening was independently performed by two researchers (ZM and GJ). The initial screening results were cross-checked for consistency, and cases with discrepant eligibility judgments requiring review by a senior orthopedic surgeon (RC) were recorded. Cohen's κ was further calculated to evaluate inter-rater agreement during case screening.

### 2.2 Establishment of the POCC framework

A structured expert-panel process was used to develop the POCC framework for TKA. The framework was not intended to predict an individual patient's actual hospitalization costs or to replace institution-specific billing and pricing procedures. The multidisciplinary panel comprised one senior and two mid-career orthopedic surgeons with extensive experience in preoperative assessment, perioperative management, and patient communication for TKA; one senior nursing specialist with expertise in perioperative nursing, rehabilitation preparation, and care management for TKA; and one senior health economist with 20 years of experience in hospital financial management and medical service pricing.

Drawing on clinical assessment frameworks, expert-review procedures used in early-stage medical artificial intelligence evaluation, and established methods for expert-consensus development (16), the research team proposed, consolidated, revised, and finalized candidate items according to their relevance to the clinical setting, identifiability from medical records, and usefulness for patient communication (17). Following a multistage expert-review approach (18), framework development comprised four stages.

In the first stage, the research team generated an initial pool of candidate items based on standard components of preoperative TKA assessment, key considerations in perioperative risk management, rehabilitation and discharge-planning needs, and patient-specific issues commonly encountered during clinical cost communication. Candidate items were required to have explicit support in the preoperative medical record, to have the potential to influence preoperative cost expectations, and to be suitable for translation into issues requiring further clinician verification or discussion with the patient. In the second stage, each panel member independently rated the clinical relevance, identifiability from medical records, and necessity for cost communication of every candidate item. Panel members did not discuss their ratings during the independent assessment. Each dimension was scored on a 4-point scale, with scores of 3 or 4 considered endorsement (19). In the third stage, the independent ratings were presented to the panel. A candidate item was required to receive endorsement from at least 80% of panel members in each of the three dimensions—clinical relevance, identifiability from medical records, and necessity for cost communication—to meet the prespecified consensus threshold (20). For items that did not meet this threshold but were considered by some panel members to have important clinical or cost-communication relevance, the panel convened a consensus meeting to revise the item definition, scope of application, and medical-record adjudication criteria. Items with overlapping content, unclear boundaries, limited relevance to cost communication, or poor identifiability from preoperative records were merged or removed. In the fourth stage, the panel conducted a final review of the revised framework items, operational definitions, medical-record adjudication criteria, and their relationship to the cost-communication setting, thereby establishing the final POCC framework. For each retained item, the item-level content validity index (I-CVI) was calculated separately for clinical relevance, identifiability from medical records, and necessity for cost communication. The I-CVI was defined as the proportion of panel members assigning a score of 3 or 4. For each dimension, the scale-level content validity index based on the average method (S-CVI/Ave) was calculated as the mean I-CVI across all retained items (21). An overall S-CVI/Ave was also calculated across all items and rating dimensions.

**Figure 1:**
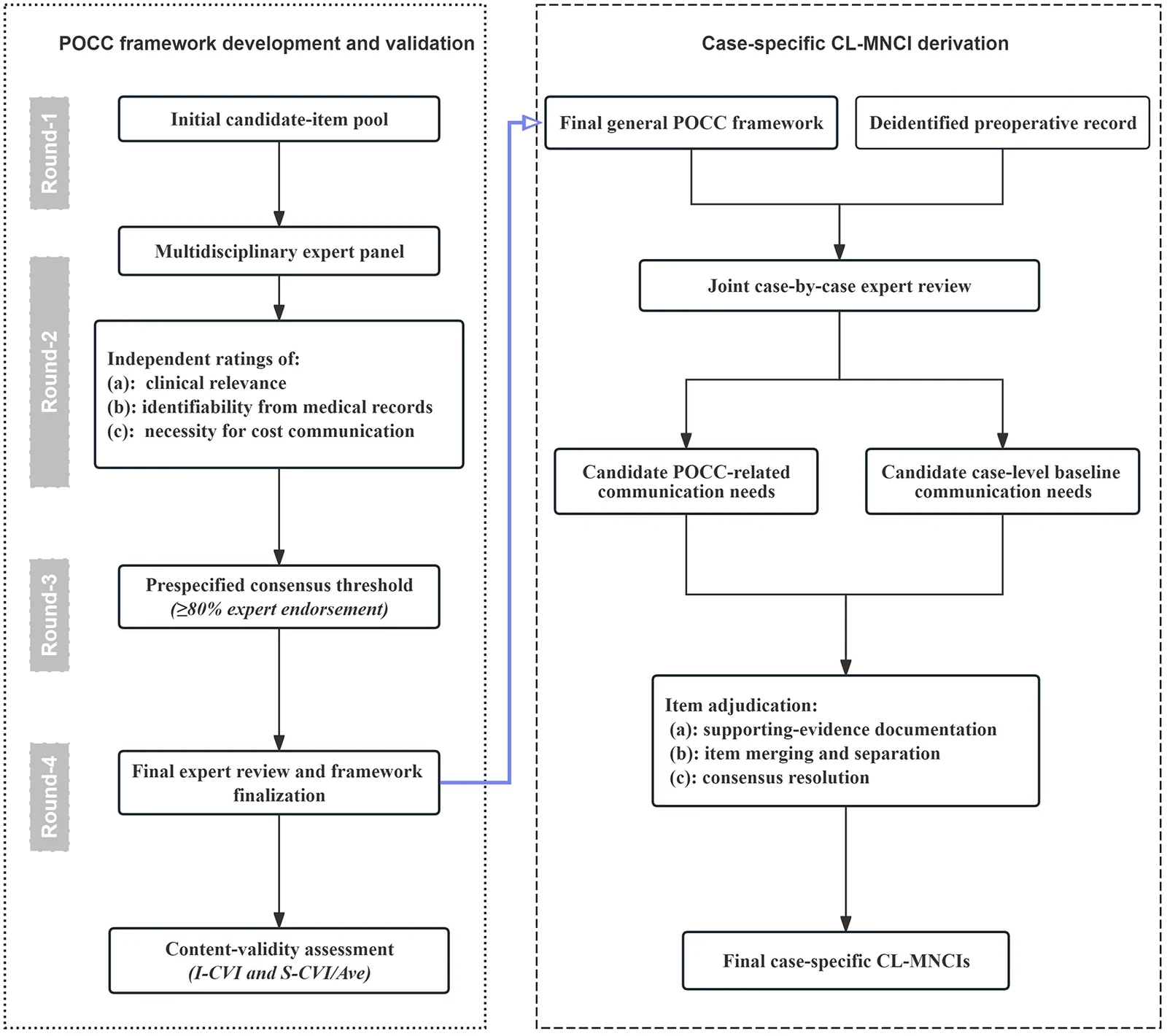
Methodological flowchart for establishment of the POCC framework and derivation of case-specific CL-MNCIs.

The final POCC framework served as the expert reference standard for identifying cost communication-related clinical cues in Task 1 and provided the conceptual scope and item domains used to develop the case-level minimum necessary communication items (CL-MNCIs). The framework represents expert consensus derived from medical-record evidence and the clinician-facing cost-communication preparation setting. It does not represent patient preferences or experiences and does not assess usability in real-world clinical workflows or patient-related outcomes. The establishment of the general POCC framework is summarized in Figure 1.

### 2.3 Establishment of CL-MNCIs

CL-MNCIs were defined as the minimum set of issues that should be prioritized for verification or discussion by clinicians during preoperative cost communication, based on the clinical conditions, treatment arrangements, and care needs documented in each patient's preoperative medical record. The CL-MNCIs served solely as the case-level reference standard for the LLM tasks in this study. All CL-MNCIs were established before any case was processed by the LLM and before any model output was evaluated. During their development, the experts were not exposed to LLM-generated content and did not consult model outputs, coverage results, or safety assessments. For each case, the experts were provided only with the deidentified preoperative medical record, the POCC framework, and the prespecified item-inclusion criteria.

An item was included as a CL-MNCI only when all of the following criteria were met: (1) the relevant clinical condition, treatment plan, payment information, or care need was explicitly supported by the patient's preoperative medical record; (2) the item could affect the patient's understanding of or preparation for cost uncertainty, additional investigations or treatments, perioperative resource use, surgical implant or material selection, rehabilitation and caregiving arrangements, or potential out-of-pocket expenses; (3) omission of the item from preoperative cost-communication preparation could leave a case-relevant and necessary communication need unaddressed; and (4) the item could be expressed as a distinct issue requiring further clinician verification, explanation, or discussion with the patient. General TKA education, content without case-specific support, issues without a clear relationship to cost communication, and content that completely duplicated the communication meaning of another item were excluded.

The expert panel jointly reviewed the anonymized preoperative medical records on a case-by-case basis and used the POCC framework to identify case-specific content potentially related to cost expectations, perioperative resource use, rehabilitation and caregiving, and patient preparation. For each candidate item, the corresponding supporting evidence in the medical record and the relevant POCC dimension were documented. Case-level basic communication needs were not triggered solely by a single positive POCC label; rather, they had to be supported by the surgical plan, uncertainty regarding the treatment approach, payment information, or rehabilitation and caregiving needs explicitly documented in the medical record. Each item was therefore determined on a case-by-case basis and was not automatically included as part of a fixed template for all TKA cases. Candidate items with different wording but the same core communication meaning were merged into a single CL-MNCI. When one item involved multiple clinical factors but served the same communication purpose, it was treated as a single item. Conversely, when the same clinical factor involved distinct issues related to resource use, cost uncertainty, or care arrangements, separate CL-MNCIs could be established. No fixed minimum or maximum number of CL-MNCIs was set for each case; the number was determined by the number of distinct necessary communication items that met the prespecified criteria. In cases of disagreement, the expert panel discussed the item on the basis of the medical-record evidence and the predefined criteria, and the final CL-MNCIs were established after consensus was reached. The final CL-MNCIs included the item text, the corresponding supporting evidence in the medical record, and the relevant evaluation dimension. They were finalized and locked before the LLM experiments began and were not subsequently added, removed, or modified on the basis of LLM-generated outputs. Because the CL-MNCIs were established through joint consensus review rather than independently generated by multiple experts, inter-rater agreement was not calculated during the initial development of the CL-MNCIs. The Cohen's κ values reported subsequently were used only to assess agreement between reviewers when semantically matching LLM-generated content to the predefined CL-MNCIs and do not represent agreement in the development of the case-level reference standard itself.

To improve transparency in the development of the reference standard while protecting patient privacy, the Supplementary material presents a representative case showing the case summary, POCC label adjudication, the medical-record evidence supporting each CL-MNCI, the final reference items, and the subsequent evaluation of the LLM-generated content. The derivation of the case-specific CL-MNCIs is summarized in Figure 1.

### 2.4 LLM task workflow and prompt design

ChatGPT-5.5 (OpenAI) was used for case-text analysis through the publicly accessible ChatGPT web interface (22). The interface did not provide a recordable identifier for the underlying model snapshot and did not disclose generation parameters such as temperature, top-p, random seed, or maximum output length. Therefore, these parameters could not be accessed or fixed by the researchers. All cases were processed using the same model option, prespecified prompts, and the platform's default generation settings.

The inputs to the LLM consisted of deidentified preoperative medical-record text. Before submission, the researchers removed patient names, hospital identification numbers, personal identification information, contact details, full addresses, exact dates, and other information that could enable direct or indirect identification. Free-text fields were also manually reviewed for residual identifying information. Only the clinical information necessary to identify cost communication-related cues and generate communication checklists was retained. Each case was processed in a separate conversation to prevent contextual information from being carried over between cases. LLM-generated outputs were used solely for the offline evaluation conducted in this study and were not used for patient cost disclosure, payment interpretation, or clinical decision-making. Access to the deidentified text and model outputs was restricted to authorized members of the research team. Only deidentified medical-record text was submitted through the public ChatGPT interface using password-protected institutional devices. This data-processing procedure was covered by the approved study protocol and informed-consent process (HN-LL-YJSLW-2026-04 and 2026Y1SKT0027-03).

The LLM experiments comprised two prespecified tasks and were conducted between May 20 and May 28, 2026. Task 1 evaluated the model's ability to identify clinical cues relevant to cost communication. For each preoperative medical record, the model assessed the presence or absence of each candidate cue category defined in the POCC framework and provided the supporting evidence for its judgment. The prompt instructed the model to base its assessment solely on the medical record provided, not to introduce labels outside the framework, and not to substitute general medical knowledge for case-specific evidence. Information that was not documented, was insufficiently supported, or was merely possible was classified as absent.

Task 2 evaluated the model's ability to generate a case-specific cost-communication checklist. Based on each patient's preoperative medical record, the model generated a checklist for clinician review and preparation before cost communication. The prompt instructed the model to generate concise items describing cost-related issues that required further verification by the clinician or discussion with the patient and to provide the corresponding supporting evidence from the medical record. The model was explicitly positioned as a clinician-facing communication preparation aid. It was instructed not to provide definitive cost estimates, health insurance reimbursement rates, or patient out-of-pocket amounts and not to replace final judgments by clinical, financial, or health insurance personnel. Task 2 did not use the outputs from Task 1, and the model was not provided with the expert-defined CL-MNCIs or the model-evaluation criteria, thereby preventing the generated content from being directly influenced by the expert reference standard. The complete prompts are provided in the Supplementary material.

### 2.5 Expert-referenced offline evaluation framework

#### 2.5.1 Task 1: evaluation of clinical cue identification for cost communication

To evaluate the LLM's ability to identify case-level clinical cues relevant to cost communication, each case–label combination was defined as a binary classification unit. Using the previously developed POCC framework, the case-level expert adjudications for the 17 categories of cost communication-related clinical cues served as the reference standard. The LLM outputs were compared with the expert adjudications for each case and each label.

A confusion matrix was constructed on the basis of these comparisons, including true positives (TP), false positives (FP), false negatives (FN), and true negatives (TN). Overall micro-precision, micro-recall, micro-F1, and accuracy were calculated. Because negative classifications predominated among the case–label units, the Matthews correlation coefficient (MCC) (23) was also calculated to provide an overall measure incorporating TP, FP, FN, and TN.

To prevent overall micro-averaged results from obscuring differences in the identification of less frequent labels, precision, recall, F1 score, and positive-class support were calculated separately for each of the 17 POCC labels. Macro-precision, macro-recall, and macro-F1 were also calculated across labels. The 95% confidence intervals for the principal overall performance measures were estimated using a case-level non-parametric bootstrap. Cases were sampled with replacement, with all 17 label classifications for each selected case retained in every resample. A total of 5,000 bootstrap resamples were performed.

#### 2.5.2 Task 2: evaluation of case-level cost-communication checklist generation and content quality

Task 2 separately evaluated the LLM's coverage of the expert-defined CL-MNCIs and the quality of all LLM-generated checklist items. CL-MNCI coverage was used to indicate whether the model conveyed the minimum necessary communication content defined by the expert panel; however, coverage alone was not considered a measure of the overall quality or clinical effectiveness of the generated checklist. Each independently generated checklist item was treated as an evaluation unit and semantically matched to the CL-MNCIs established for the corresponding case. A match was determined by whether the generated item conveyed the core communication meaning of a CL-MNCI, rather than by identical wording or sentence structure. When multiple generated items matched the same CL-MNCI, that CL-MNCI was counted as covered only once. When a single generated item clearly conveyed multiple distinct CL-MNCIs, coverage of each corresponding CL-MNCI was recorded separately. Accordingly, the number of generated items and the number of covered CL-MNCIs were treated as distinct evaluation units and reported separately. The overall CL-MNCI coverage rate, case-level coverage rate, and proportion of cases in which all CL-MNCIs were covered were calculated.

All generated items were first screened for safety events. Items meeting the predefined criteria for a safety event were assigned preferentially to the safety evaluation and were excluded from the general content-quality classification. For generated items not classified as safety events, reviewers first determined whether each item semantically matched one or more CL-MNCIs. Non-safety generated items that did not match any CL-MNCI were further classified as: (1) case-relevant supplementary content supported by the medical record; (2) case-relevant but redundant content; or (3) content with insufficient support from the medical record. The numbers and proportions of all generated items, covered CL-MNCIs, safety events, and each category of additional generated content were reported. The proportion of case-relevant, record-supported generated items was calculated as the sum of generated items matching CL-MNCIs, record-supported supplementary items, and redundant items, divided by the total number of generated items. This measure was interpreted together with the findings for redundant content, content with insufficient record support, and safety events and was not regarded as a standalone measure of checklist precision or clinical effectiveness.

#### 2.5.3 Safety evaluation of LLM-generated content

A safety event was defined as generated content that could cause patient misunderstanding, create unreasonable cost expectations, or exceed the role boundaries of a clinician-facing communication preparation aid. Safety events included fabricated medical-record information, unsupported inferences, misleading reassurance, and inappropriate role boundaries. A hierarchical classification approach was applied. Once an item met the definition of a safety event, it was assigned to the corresponding safety category and was not included in the general content-quality classification. If an item potentially met the criteria for more than one safety category, its primary category was determined according to the predominant potential risk. Safety events were summarized both by generated item and by case. A case was considered to contain a safety event when at least one generated item met the safety-event definition.

#### 2.5.4 Human review process and inter-rater agreement analysis

Two researchers (LK and HF) independently evaluated all LLM-generated checklist items according to prespecified adjudication criteria. Specifically, each generated item was assessed for semantic matching to the predefined CL-MNCIs, classification of additional generated content, and identification of safety events. Before the formal evaluation, both reviewers underwent standardized training using example items. The evaluation was conducted in the following sequence: safety events were identified first; items not classified as safety events were then assessed for whether they matched a CL-MNCI; and non-safety items that did not match a CL-MNCI were subsequently classified as additional generated content. Percentage agreement and Cohen's κ were calculated separately for: (1) semantic matching of LLM-generated items to the predefined CL-MNCIs; (2) classification of unmatched non-safety generated items; (3) identification of safety events; and (4) classification of safety-event types. Disagreements were first resolved through discussion between the two reviewers. When consensus could not be reached, a third senior surgeon (CY) reviewed the item and made the final adjudication. Further details are provided in the Supplementary material.

### 2.6 Repeated-generation stability assessment

To evaluate the stability of LLM outputs under the same task conditions (24), this study conducted an exploratory repeated generation stability analysis (25). Given the workload required for repeated generation and manual evaluation across the full cohort, and with reference to the sample designs used in previous reproducibility studies (26), 24 cases were selected for analysis, representing 30.0% of the full cohort. Based on the number of expert-defined CL-MNCIs per case, the cases were stratified into three groups containing 5, 6–8, and 9–10 CL-MNCIs, respectively. According to the distribution of these strata in the full cohort, 2, 19, and 3 cases were selected non-randomly in the order of their prespecified deidentified case numbers. Case selection was completed before the additional repeated-generation runs and was not based on model performance in terms of CL-MNCI coverage or safety-event outcomes. This stratification was used solely to cover different ranges of case-level communication-item counts and did not represent overall clinical complexity or communication complexity.

The initial outputs were defined as Run 1. Under identical conditions with respect to the input case text, task prompts, and experimental environment, two additional independent generations were performed and defined as Run 2 and Run 3, respectively. These runs were completed on May 30 and May 31, 2026. Each run was conducted in a new, independent conversation, and the model was not provided with outputs from any other run. For Task 1, micro-precision, micro-recall, and micro-F1 were compared across the three runs, and the numbers and proportions of discordant case–label classifications were recorded. For Task 2, the sets of CL-MNCIs covered in each run were compared, pairwise Jaccard similarity coefficients were calculated, and the proportions of CL-MNCIs covered in all three runs, in two runs, in one run, or in none of the runs were determined. The safety analysis compared the occurrence of safety events and agreement in the primary safety-event categories across runs. This analysis was intended to describe cross-run variation in both aggregate performance measures and specific output content; no inferential statistical testing was performed.

## 3 Results

### 3.1 Case selection

**Figure 2:**
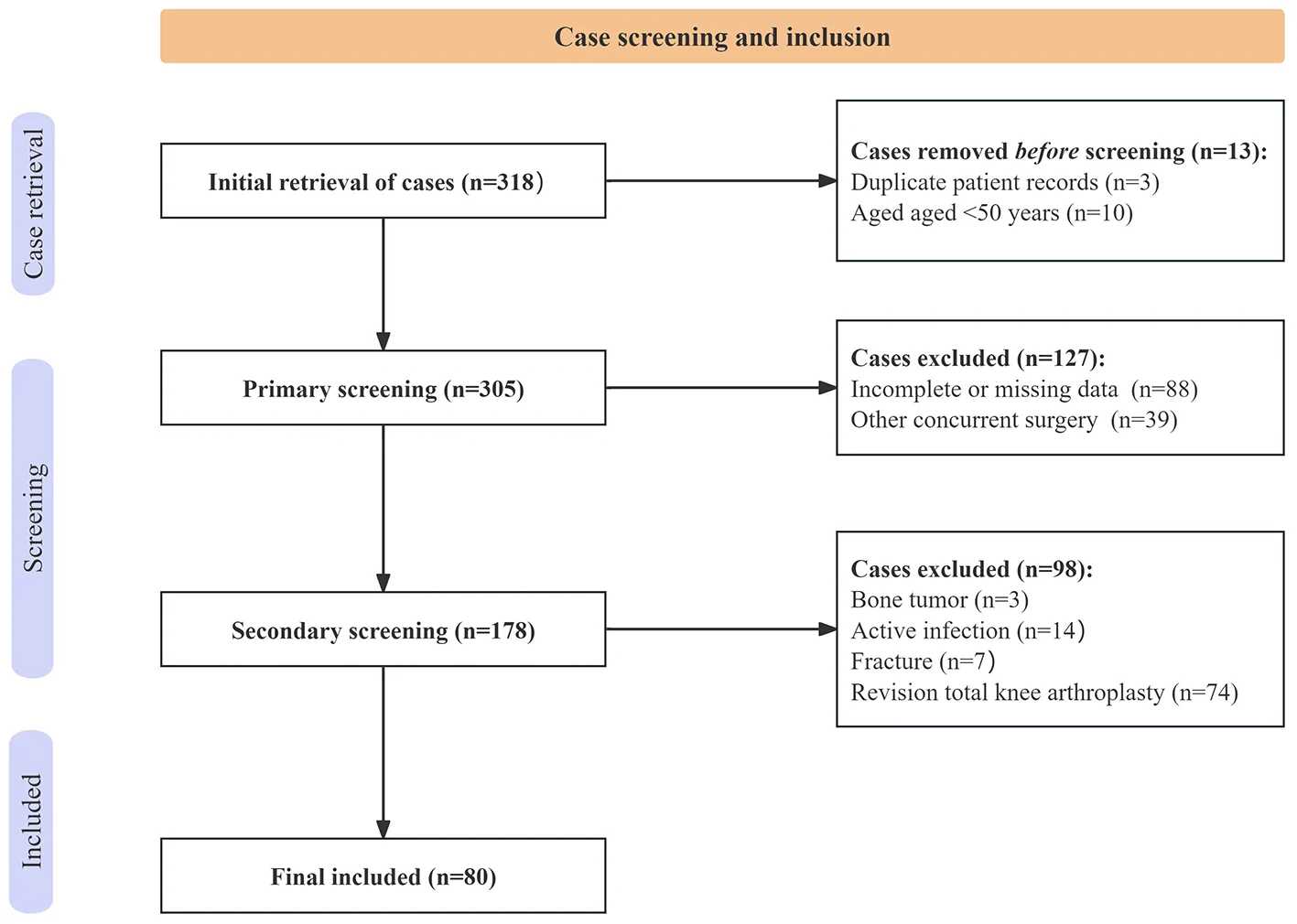
Flowchart of the study selection process.

A total of 318 medical records were obtained from the two hospitals. 13 records were removed before formal screening, and 225 records were excluded during the formal screening process, leaving 80 medical records for final inclusion. The screening process is shown in Figure 2. Among the 305 records entering initial independent screening, the two researchers reached consistent judgments for 291 records, including 76 records judged eligible for inclusion, with 14 initial discrepancies (initial agreement rate, 95.4%; Cohen's κ, 0.884), indicating high inter-rater agreement during case screening. The discrepancies mainly involved the completeness of preoperative data, whether the case represented revision TKA, and diagnostic classification. After review by a senior orthopedic surgeon, 10 discrepant records were finally excluded and 4 were included. All patient identifying information in the included medical records was anonymized. Among the included patients, the mean age was 70.74 ± 5.29 years. There were 26 male and 54 female patients. The surgical side was the left knee in 38 cases and the right knee in 42 cases. Patient baseline characteristics are provided in the Supplementary data S1.

### 3.2 Development of the POCC framework and content validity assessment

Using a structured expert-panel process, the research team initially generated 24 candidate items related to cost communication. Following consensus discussion, 21 items met the prespecified consensus criteria. Three items—preoperative pain severity, radiographic severity of degeneration, and medication allergy history—were removed because of their limited relevance to the cost-communication context or because they could not be identified reliably from preoperative medical records. In addition, eight candidate items were consolidated into four items. Specifically, smoking history and alcohol use history were combined as smoking and alcohol use; a history of diabetes or use of glucose-lowering medication and abnormal glycemic control were combined as abnormal glycemic status; anticoagulant or antiplatelet therapy and coagulation abnormalities or thrombotic risk were combined as antithrombotic therapy-related risk; and anemia and hypoalbuminemia or malnutrition were combined as abnormal nutritional status. The final POCC framework comprised four dimensions and 17 clinical cues relevant to cost communication, covering baseline patient characteristics, perioperative management risks, local complexity factors, and preoperative functional status. The framework served as the expert reference standard for Task 1 and was also used to establish the CL-MNCIs for Task 2, as shown in Table 1.

**Table 1:** Expert-derived framework for TKA preoperative cost communication.

Category	Cue label	Identification criterion	Minimum necessary communication item
Baseline patient characteristics	Advanced age	Age ≥75 years	Indicate that the need for preoperative assessment, anesthesia assessment, and postoperative observation may be increased; discuss rehabilitation and care arrangements in advance.
	Elevated BMI	BMI ≥28 kg/m^2^	Indicate that perioperative nursing, wound management, infection prevention, and rehabilitation implementation may require additional support.
	Smoking and alcohol consumption	Smoking or alcohol consumption history	Confirm the recent status; indicate the need for preoperative optimization, perioperative monitoring, wound management, and attention to the risk of delayed recovery.
Perioperative management risks	Abnormal glycemic status	History of diabetes mellitus or current use of glucose-lowering medications	Address preoperative glycemic optimization, perioperative glucose monitoring, infection prevention, and wound-care needs; explain that inadequate glycemic control may require adjustment of the surgical schedule.
	Cardiovascular disease	Coronary artery disease, arrhythmia, history of myocardial infarction, or cardiac insufficiency	Address the need for preoperative cardiac evaluation, specialist consultation, and perioperative monitoring; severe cases may require postoperative observation or monitored care
	Hypertension	History of hypertension, poorly controlled blood pressure, or current use of antihypertensive medications	Address preoperative blood pressure optimization and medication adjustment; marked blood pressure fluctuations may affect surgical scheduling and postoperative monitoring.
	Renal dysfunction	Renal insufficiency or abnormal creatinine/eGFR	Explain that renal function may affect medication selection, examination planning, and fluid management; discuss renal function reassessment, specialist evaluation, and postoperative monitoring when necessary.
	Pulmonary disease	COPD, asthma, chronic bronchitis, pulmonary infection, or abnormal pulmonary function	Address anesthesia-related and postoperative respiratory management risks; discuss pulmonary function assessment, respiratory therapy, oxygen therapy, or postoperative respiratory observation when necessary.
	Infection risk	Suspected infection, skin breakdown, or abnormal inflammatory markers	Address the need for preoperative infection screening and infection control, including possible surgical postponement; discuss postoperative wound care, antimicrobial treatment, or additional intervention if needed.
	Antithrombotic therapy-related risk	Abnormal coagulation function, anticoagulant or antiplatelet therapy, or risk of DVT/PE	Address medication discontinuation, bridging therapy, coagulation reassessment, and transfusion preparation; discuss perioperative bleeding risk and postoperative thromboprophylaxis
	Abnormal bone mineral density	Osteoporosis or osteopenia	Address potential implications for prosthesis fixation, perioperative fracture risk, and rehabilitation safety; discuss anti-osteoporosis treatment, fall prevention, and cautious rehabilitation when appropriate.
	Abnormal nutritional status	Anemia, hypoalbuminemia, malnutrition, or decreased albumin level	Address preoperative correction of anemia, nutritional support, and transfusion assessment; severe abnormalities may affect surgical scheduling and postoperative recovery.
Local complexity factors	Severe deformity	Severe varus or valgus deformity, flexion contracture, or other marked knee deformity	Discuss the potential impact on surgical difficulty, implant or instrument preparation, operative time, and postoperative rehabilitation needs.
	History of previous knee surgery	Prior surgery on the affected knee	Address the possible impact of previous surgery on surgical complexity, soft-tissue management, infection risk, implant selection, and postoperative recovery
	Non-primary osteoarthritis	Knee osteoarthritis secondary to trauma, inflammatory disease, infection, or other non-primary causes	Explain that the underlying etiology may increase surgical complexity and uncertainty in implant or material requirements; discuss possible effects on perioperative planning and cost expectations.
Preoperative functional status	Poor preoperative mobility	Marked limitation in walking ability or daily activities before surgery	Discuss rehabilitation difficulty, postoperative care needs, discharge planning, and possible requirements for extended rehabilitation support.
	Ambulation with assistive devices	Preoperative use of a cane, walker, crutches, wheelchair, or other assistive devices for ambulation	Address the need for postoperative mobility support, assistive-device preparation, fall prevention, caregiver involvement, and rehabilitation or discharge arrangements.

Expert content-validity assessment showed that the I-CVI values of the 17 labels ranged from 0.80 to 1.00 across the three evaluation domains, and all items met the predefined retention criteria. The S-CVI/Ave values for clinical relevance, identifiability from medical records, and necessity for cost communication were 0.976, 0.953, and 0.965, respectively, with an overall S-CVI/Ave of 0.965, indicating good content validity of the framework. Detailed results are provided in the Supplementary data S1.

### 3.3 Performance in identifying clinical cues relevant to cost communication

Using the POCC framework, the LLM performed closed-set identification across 80 preoperative TKA records, yielding 1,360 case–label classification units. With the expert annotations as the reference standard, the LLM produced 300 true positives, 33 false positives, 38 false negatives, and 989 true negatives. Overall, the model demonstrated good classification performance in identifying clinical cues relevant to cost communication. Micro-precision was 0.901 (95% CI, 0.876–0.925), micro-recall was 0.888 (95% CI, 0.849–0.924), micro-F1 was 0.894 (95% CI, 0.872–0.915), and overall accuracy was 0.948 (95% CI, 0.938–0.957). Given the predominance of negative classifications, the MCC was additionally calculated and was 0.860 (95% CI, 0.831–0.887).

To reduce the influence of more frequent labels on the micro-averaged results, macro-precision, macro-recall, and macro-F1 were calculated across the 17 labels and were 0.853, 0.830, and 0.840, respectively. These values were lower than the corresponding overall micro-averaged results, indicating variation in performance for some less frequent cost communication-related cues. Advanced age and elevated BMI were identified without error, with an F1 score of 1.000 for both labels. The F1 scores for severe knee deformity and previous knee surgery were 0.987 and 0.900, respectively. Performance was lowest for non-primary osteoarthritis, with a precision of 0.400, recall of 0.500, and F1 score of 0.444. The F1 scores for antithrombotic therapy-related risk, infection risk, and long-term use of assistive devices for ambulation were 0.727, 0.750, and 0.750, respectively, as shown in Table 2.

**Table 2:** LLM performance in identifying expert-defined cost-communication cues.

Clinical cue	Positive support, *n*	Precision	Recall	F1-score
Advanced age	15	1.000	1.000	1.000
Elevated BMI	56	1.000	1.000	1.000
Smoking and alcohol consumption	26	0.833	0.769	0.800
Abnormal glycemic status	19	0.889	0.842	0.865
Cardiovascular disease	14	0.857	0.857	0.857
Hypertension	43	0.952	0.930	0.941
Renal dysfunction	8	0.857	0.750	0.800
Pulmonary disease	8	0.857	0.750	0.800
Infection risk	8	0.750	0.750	0.750
Antithrombotic therapy-related risk	6	0.800	0.667	0.727
Abnormal bone mineral density	23	0.955	0.913	0.933
Abnormal nutritional status	15	0.867	0.867	0.867
Severe deformity	39	0.975	1.000	0.987
History of previous knee surgery	10	0.900	0.900	0.900
Non-primary osteoarthritis	8	0.400	0.500	0.444
Poor preoperative mobility	28	0.857	0.857	0.857
Long-term use of assistive devices for ambulation	12	0.750	0.750	0.750
Micro-average	338	0.901	0.888	0.894
Macro-average	–	0.853	0.830	0.840

### 3.4 Case-level cost-communication checklist generation and content quality assessment

Across the 80 cases, the expert panel established 569 case-level CL-MNCIs, with a median of 7 items per case (IQR, 6–8). The LLM generated 716 checklist items, with a median of 9 items per case (IQR, 8–10). Of the 569 CL-MNCIs, 483 were covered by the generated content, corresponding to an overall coverage rate of 84.9% (483/569). The median case-level coverage rate was 85.7% (IQR, 80.0%−88.9%). Complete coverage of all CL-MNCIs was achieved in 17 cases (21.3%); 44 cases had one uncovered CL-MNCI, 15 had two uncovered CL-MNCIs, and 4 had three uncovered CL-MNCIs. Among the 86 uncovered CL-MNCIs, 40 (46.5%) reflected insufficient translation of perioperative management issues into communication content, 21 (24.4%) reflected insufficient specification of rehabilitation- and caregiving-related cost communication, 18 (20.9%) concerned uncertainty regarding the surgical plan and associated costs, and 7 (8.1%) involved inadequate verification of payment or settlement arrangements. The categories of uncovered content are summarized in Table 3.

**Table 3:** Categories of uncovered expert-defined CL-MNCIs in LLM-generated communication checklists.

Coverage gap category	Uncovered items, *n*/*N* (%)	Examples of unaddressed content
Perioperative management implications	40/86 (46.5%)	1. Lack of cost communication regarding glycemic control, including preoperative optimization, infection prevention, glucose monitoring, and medication adjustment. 2. Lack of cost communication on anticoagulation-related specialist consultation and perioperative management. 3. Insufficient indication of possible corrective treatment, nutritional support, transfusion assessment, or treatment timeline changes related to anemia. 4. Insufficient indication of possible infection control, dressing changes, anti-infective treatment, or surgical delay.
Rehabilitation and care resources	21/86 (24.4%)	1. Lack of communication about transportation and home adaptation costs for patients with follow-up difficulties. 2. Lack of communication about additional costs for rehabilitation services and assistive devices.
Surgical-plan uncertainty	18/86 (20.9%)	1. Insufficient communication about cost implications of surgical complexity, material preparation, intraoperative management, and prosthesis selection. 2. Insufficient communication that the final plan requires confirmation through preoperative assessment and intraoperative judgment.
Institutional payment verification	7/86 (8.1%)	1. Lack of communication that additional consultations or special materials require cost confirmation according to institutional procedures.

In accordance with the prespecified hierarchical classification approach, all 716 LLM-generated items were first screened for safety events. Twelve items were classified as safety events and occurred in 9 cases (11.3%). These included 2 items containing fabricated medical-record information, 4 containing unsupported inferences, 3 containing misleading reassurance, and 3 involving inappropriate role boundaries, as shown in Table 4. Among the remaining 704 non-safety generated items, 483 semantically matched the expert-defined CL-MNCIs, whereas 221 did not match any CL-MNCI. The unmatched items comprised 132 items of case-relevant supplementary content supported by the medical record, 50 case-relevant but redundant items, and 39 items with insufficient support from the medical record. A total of 665 generated items either matched a CL-MNCI, represented record-supported supplementary content, or were redundant but record-supported, accounting for 92.9% of all generated items (665/716). Notably, both the number of generated items matching CL-MNCIs and the number of covered CL-MNCIs were 483; however, these represented distinct evaluation units. The former was based on individual LLM-generated items, whereas the latter was based on the expert-defined CL-MNCIs.

**Table 4:** Safety events in LLM-generated preoperative cost communication checklists for TKA.

Category	Events, *n*/*N*	Medical record context	LLM output	Safety rationale
Fabricated medical record information	2/12 (16.7%)	Family member information not documented	Insufficient family care support; caregiver or rehabilitation facility recommended.	May lead physicians to discuss care and costs based on an incorrect premise.
		Payment or insurance coverage not documented	Possible financial constraints; cost burden should be discussed in advance	Treats undocumented payment information as a patient-specific financial risk
Unsupported inferences	4/12 (33.3%)	Planned implant type and cost information not documented	Routine costs are unlikely to increase substantially	Provides a definite estimate of implant-related costs without a documented implant plan
		Additional workup for comorbidities not documented	Additional preoperative tests are generally low-cost	Downplays the potential cost impact of additional comorbidity-related assessment
Misleading reassurance	3/12 (25.0%)	Severe knee deformity and functional limitation documented	Additional cost increases are unlikely	Understates potential cost uncertainty despite indicators of severe disease
		Multiple comorbidities documented	Routine perioperative management is sufficient	Underestimates cost implications of multiple comorbidities, risking clinician–patient mistrust
Inappropriate role-boundary content	3/12 (25.0%)	Planned implant type and cost information not documented	The patient should choose a more economical implant option	Cost-driven implant recommendation may interfere with shared decision-making

Inter-rater agreement was 97.3% for semantic matching of LLM-generated items to CL-MNCIs, with a Cohen's κ of 0.939. Agreement was 93.2% for classification of unmatched non-safety generated items (κ = 0.879), 99.6% for identification of safety events (κ = 0.878), and 91.7% for classification of safety-event types (κ = 0.887). Further details are provided in the Supplementary material.

### 3.5 Repeated-generation stability

The repeated-generation stability analysis included 24 cases, representing 30.0% of the full cohort. Of these, 2, 19, and 3 cases contained 5, 6–8, and 9–10 CL-MNCIs, respectively. The analysis included 180 expert-defined CL-MNCIs and 408 case–label classification units. For Task 1, micro-precision across Runs 1, 2, and 3 was 0.917, 0.900, and 0.927, respectively; micro-recall was 0.871, 0.891, and 0.881; and micro-F1 was 0.893, 0.896, and 0.904. Pairwise agreement in case–label classifications was 96.6% between Runs 1 and 2, 96.6% between Runs 1 and 3, and 95.1% between Runs 2 and 3. Of the 408 classification units, 24 (5.9%) yielded non-identical classifications across the three runs. For Task 2, Runs 1, 2, and 3 covered 149, 151, and 150 CL-MNCIs, corresponding to coverage rates of 82.8%, 83.9%, and 83.3%, respectively. The median case-level coverage rate was 85.7% in all three runs, with IQRs of 75.0%−87.5%, 75.0%−87.5%, and 75.0%−89.2%, respectively. The pairwise Jaccard similarities between the sets of covered CL-MNCIs were 0.818 for Runs 1 and 2, 0.823 for Runs 1 and 3, and 0.813 for Runs 2 and 3. The corresponding agreement rates for CL-MNCI coverage status were 83.3%, 83.9%, and 82.8%, respectively.

Among the 180 CL-MNCIs, 126 (70.0%) were covered in all three runs, 27 (15.0%) were covered in two runs, 18 (10.0%) were covered in only one run, and 9 (5.0%) were not covered in any run. Safety events occurred in 3 cases (12.5%) in Run 1, 4 cases (16.7%) in Run 2, and 3 cases (12.5%) in Run 3. Pairwise agreement in case-level safety-event classification was 87.5%, 91.7%, and 95.8%, respectively. Safety-event classifications were not identical across all three runs in 3 cases (12.5%). Although the aggregate performance measures were similar across the three runs, direct agreement analyses showed that specific label classifications, the sets of covered CL-MNCIs, and case-level safety-event classifications were not completely stable, as shown in Table 5.

**Table 5:** Direct output stability across three repeated generations.

Stability measure	Run 1 vs. Run 2	Run 1 vs. Run 3	Run 2 vs. Run 3	Overall cross-run result
Task 1 label-level agreement, *n*/*N* (%)	394/408 (96.6%)	394/408 (96.6%)	388/408 (95.1%)	24/408 decisions (5.9%) were not identical across all three runs
Task 2 CL-MNCI Jaccard similarity	0.818	0.823	0.813	Mean pairwise Jaccard similarity: 81.8%
Task 2 CL-MNCI coverage-status agreement, *n*/*N* (%)	150/180 (83.3%)	151/180 (83.9%)	149/180 (82.8%)	Agreement included both jointly covered and jointly uncovered CL-MNCIs
Case-level safety-classification agreement, *n*/*N* (%)	21/24 (87.5%)	22/24 (91.7%)	23/24 (95.8%)	Safety classification was not identical across all three runs in 3/24 cases (12.5%)

## 4 Discussion

### 4.1 Principal findings

Using real-world preoperative TKA records from two hospitals, this study conducted an expert-referenced offline evaluation of an LLM's performance in identifying clinical cues relevant to cost communication and generating clinician-facing preoperative cost-communication preparation checklists. We further assessed the quality of the generated content, potential safety events, and substantive output stability across repeated runs. Current research on TKA costs has highlighted the importance of cost-related issues from multiple perspectives, including patient out-of-pocket expenses, regional differences (27), insurance payer types (2), the impact of complications (28), and prediction of length of hospital stay (29). However, relatively limited attention has been paid to how clinicians can translate patient-specific clinical risks into discussable cost preparation points before surgery. With the expanding use of LLMs in medicine, recent orthopedic studies have begun to evaluate their performance in orthopedic disease knowledge generation (12), patient education material generation, and clinical guideline concordance (14), suggesting the potential value of LLMs in orthopedic information support. At the same time, applications of LLMs in health economics are expanding beyond medical text processing toward areas such as data extraction for cost-effectiveness analysis, quality assessment of economic evaluations, and prediction of healthcare cost risks (30). However, existing studies continue to emphasize the need for human oversight and methodological validation, indicating that LLMs cannot yet directly replace cost-related judgments in specific clinical scenarios (31).Therefore, unlike previous studies that have mainly focused on medical knowledge question answering, patient education, or clinical decision support (32), the present study defined preoperative cost communication for TKA as a clinician-facing, review-dependent preparation task. We specifically examined whether the LLM could identify cost-relevant cues from preoperative medical records and generate case-level communication checklists that clinicians could supplement, remove, or revise. Overall, the model identified and covered most expert-defined communication content. However, omissions of necessary content, variation in the quality of additional generated content, safety risks, and cross-run variability remained. These findings suggest that the model is better suited to preliminary information organization before clinician review than to directly providing cost information to patients or replacing professional cost-related judgment.

### 4.2 Cost-communication cue framework and identification performance

This study categorized cost communication cues into four domains: patient baseline characteristics, perioperative management risks, local complexity, and preoperative functional status. The 17 cost communication cue labels established in this study were not intended to predict the specific hospitalization costs of individual patients. Rather, they were designed to identify clinical information in preoperative medical records that may be relevant to POCC preparation, indicating potential needs for additional perioperative management, surgical preparation, rehabilitation and care planning, or communication about cost expectations. During framework development, preoperative pain severity, radiographic degeneration severity, and medication allergy history were not included in the final framework. The former two items more directly reflect surgical indications and disease severity in TKA and are difficult to translate independently into distinct cost communication points. Medication allergy history may be relevant to cost communication only when considered in relation to specific contexts, such as medication substitution, anesthetic management, or implant/material selection. Therefore, the final framework emphasized clinical cues that could be reliably identified from preoperative medical records and translated into clinician-facing cost communication preparation content. Previous studies have shown that patient baseline characteristics, health behaviors, and modifiable risk factors, such as obesity, diabetes, anemia, and malnutrition, may affect perioperative optimization, infection prevention, complication risk, and postoperative recovery (33). Comorbidities such as cardiovascular disease, hypertension, renal dysfunction, pulmonary disease, infection risk, and antithrombotic therapy may involve additional assessments, specialist consultations, perioperative monitoring, medication adjustment, or changes in surgical scheduling (34). Meanwhile, local complexity factors, including severe deformity, previous knee surgery, and non-primary osteoarthritis, may affect surgical difficulty, implant or material preparation, and intraoperative management (35). Poor preoperative mobility, long-term use of assistive devices for ambulation, and insufficient rehabilitation support are closely related to discharge planning, rehabilitation resources, and care needs (36).

The Task 1 results showed that the LLM was generally able to identify most of the expert-defined clinical cues relevant to cost communication, with a micro-F1 of 0.894. The MCC was 0.860 and remained high when true positives, false positives, true negatives, and false negatives were considered simultaneously, indicating that the overall performance was not driven solely by the large number of negative classifications. However, the macro-F1 was 0.840, which was lower than the micro-F1 and indicated uneven performance across individual labels. The LLM more readily identified relatively explicit or structured information, including advanced age, elevated BMI, smoking or alcohol use, severe knee deformity, and previous knee surgery. In contrast, performance was lowest for non-primary osteoarthritis, with an F1 score of 0.444. The F1 scores for antithrombotic therapy-related risk, infection risk, and long-term use of assistive devices for ambulation were 0.727, 0.750, and 0.750, respectively. These labels had relatively few positive cases, and their classification often required integration of medical history, medication records, negated statements, and functional information rather than reliance on a single explicit field. These findings indicate that favorable overall classification performance cannot replace label-specific evaluation. The LLM may produce more reliable classifications for cues supported by explicit thresholds or structured evidence, whereas cues based on dispersed evidence, exclusion of alternative etiologies, or contextual interpretation may remain susceptible to under-identification or overidentification. Therefore, in clinician-facing cost-communication preparation, LLM-based cue identification should be used to support the organization of medical-record information rather than to replace clinician review of the original records, medication status, and treatment plan. Human verification should be retained, particularly for infrequent or boundary-sensitive cues such as antithrombotic management, infection risk, the etiology of secondary osteoarthritis, and the need for functional assistance.

### 4.3 Translating clinical cues into cost-communication items

The potential value of a clinician-facing cost-communication checklist lies not in simply listing risks documented in the medical record, but in helping clinicians identify, in advance, communication issues that may affect patients' cost expectations and preoperative preparation and that require further verification or explanation. Effective cost communication should be grounded in the specific treatment pathway and payment context rather than limited to generic statements about costs (4). In this study, CL-MNCIs served as the expert reference standard for the minimum necessary communication content at the case level. The LLM covered most expert-defined items, but complete coverage was achieved in only a subset of cases. This finding suggests that the model can generate a reasonably structured draft for communication preparation but cannot yet ensure that all necessary communication content is identified for every patient. The categories of uncovered CL-MNCIs further illustrate the distinction between identifying clinical cues and translating those cues into cost-communication items. The most common omissions involved perioperative management. For example, the model might identify abnormal glycemic status, hypertension, anemia, infection risk, or a history of antithrombotic therapy but fail to explain that these conditions could entail preoperative optimization, medication adjustment, repeat testing, specialist consultation, enhanced monitoring, or changes in treatment timing, all of which may have implications for resource use and cost communication. Other omissions concerned rehabilitation and caregiving resources, uncertainty regarding the surgical plan or material use, and institution-specific payment or settlement verification. Thus, identifying a clinical risk does not necessarily mean that the risk has been translated into a specific and clearly bounded cost-communication item. Evaluation of all 716 generated items also showed that CL-MNCI coverage alone cannot represent the overall quality of the generated checklist. In addition to items matching the expert reference standard, the model generated case-relevant supplementary content supported by the medical record, redundant content, and content with insufficient record support. Record-supported supplementary content indicates that CL-MNCIs, as a minimum necessary reference standard, do not preclude the model from generating other reasonable communication-preparation suggestions. However, redundant content may increase the clinician's burden of reviewing and revising the checklist, whereas insufficiently supported content suggests that the model may overinterpret general possibilities as case-specific conclusions. The reported proportion of case-relevant, record-supported generated items included items matching CL-MNCIs, reasonable supplementary items, and redundant items. It should therefore be interpreted as a broad measure of content support rather than as precision in the strict statistical sense, and it does not directly reflect checklist conciseness, efficiency, or clinical effectiveness. Accordingly, evaluation of clinician-facing cost-communication checklists should consider CL-MNCI coverage, case-specific record support, reasonable supplementary content, redundancy, and safety risks together. Neither the 84.9% coverage of necessary content nor the 92.9% proportion of record-supported content alone is sufficient to determine that the model output is complete or ready for direct use. Taken together, these findings suggest that the LLM is better suited to preliminary information organization during cost-communication preparation. The generated checklist still requires clinicians to supplement, remove, and revise items in light of the original medical record, the actual treatment plan, and institution-specific payment policies.

### 4.4 Safety and the need for clinician review

In medical settings, the safety issues of LLMs cannot be overlooked. Previous evaluation studies of medical LLMs have proposed that manual assessment should consider dimensions such as factuality, reasoning, bias, and possible harm (16). The World Health Organization guidance on the ethics and governance of large multimodal models in health notes that generative AI may produce false, inaccurate, biased, or incomplete information in healthcare settings and may also induce automation bias (37). In the present study, safety involved not only the factual accuracy of medical information, but also whether the model adhered strictly to the current medical record and established treatment plan, appropriately conveyed uncertainty regarding costs and payment, and remained within the role boundaries of a clinician-facing communication preparation aid. Safety events were therefore distinguished from general omissions, redundancy, and insufficiently supported content and were identified before the general content-quality classification. Some generated content may primarily affect checklist quality when it lacks adequate support but merely represents an inaccurate general suggestion. However, when such content could alter a clinician's or patient's understanding of treatment, payment responsibility, or financial risk, it carries more direct safety implications. For example, presenting an undetermined implant or material cost as a fixed outcome, or asserting that severe deformity would generally not increase costs, could create inappropriate cost expectations. Accordingly, we applied a hierarchical and mutually exclusive classification pathway: once an item was classified as a safety event, it was not simultaneously assigned to a general content-quality category and was not counted toward CL-MNCI coverage. Although safety events accounted for only 1.7% of all generated items, they occurred in 11.3% of cases, indicating that a low item-level frequency does not imply negligible potential clinical impact. Fabricated medical-record information may incorrectly introduce undocumented diseases, care needs, or payment difficulties as a basis for communication. Unsupported inferences may present definitive conclusions before insurance coverage, out-of-pocket proportions, additional investigations, or material costs have been verified. Misleading reassurance may understate genuine uncertainty related to the surgical plan, comorbidity management, or rehabilitation arrangements. Inappropriate role boundaries may lead the model to move beyond information organization and make unsuitable recommendations regarding implant selection, treatment plans, or cost commitments. Inter-rater agreement was 99.6% for safety-event identification and 91.7% for safety-event subtype classification, with Cohen's κ values of 0.878 and 0.887, respectively, indicating that the prespecified safety criteria were operationally applicable. However, these findings do not indicate that the model is sufficiently safe for direct patient-facing cost communication. A more appropriate use would be for the LLM to generate an initial communication-preparation draft, followed by item-by-item clinician verification against the original medical record and actual treatment plan. Information concerning insurance coverage lists, settlement procedures, reimbursement rates, and estimated out-of-pocket costs should be further confirmed by hospital insurance, billing, or financial-management personnel. Human review is therefore not merely an additional step to optimize model output, but an essential component of maintaining the safety boundaries of this application.

### 4.5 Substantive output variability across repeated generations

LLM outputs are inherently subject to a degree of randomness and uncertainty. Even when the same input, model, and controllable operating conditions are maintained, repeated generations may not produce identical results (38). Stability evaluation is therefore an important step before the deployment of medical LLMs. Previous studies have used repeated questioning or repeated generation designs to evaluate LLMs in scenarios such as medical examination question answering, triage, and diagnostic reasoning (39). Test cases should cover clinically meaningful variation to avoid overall metrics masking performance differences across subgroups (40). In this study, cases for repeated generation were selected according to the number of expert-defined CL-MNCIs per case, thereby covering a range of 5–10 communication items within the full cohort. This stratification reflected the number of case-level communication items rather than the patient's overall clinical complexity. Similar aggregate performance across runs indicates only that average performance was comparable; it does not establish that individual label classifications or generated content were consistent. Across the three runs, the aggregate performance measures for Task 1 were similar, but pairwise agreement for the 408 case–label classification units ranged from 95.1 to 96.6%, indicating that a small proportion of clinical-cue classifications varied across runs. Content variability was more evident for Task 2. Pairwise Jaccard similarity for the sets of covered CL-MNCIs ranged from 0.813 to 0.823, indicating substantial overlap across runs but incomplete consistency in the specific items covered. Of the 180 CL-MNCIs, 126 (70.0%) were covered in all three runs, whereas 54 (30.0%) were not consistently covered. These included 27 items (15.0%) covered in two runs, 18 (10.0%) covered in only one run, and 9 (5.0%) not covered in any run. Thus, even when overall coverage rates were similar, the model could omit different necessary communication items across runs.

Such variability may extend beyond differences in wording and may affect whether clinical information is translated into specific communication issues concerning repeat testing, medication adjustment, rehabilitation resources, or uncertainty regarding material-related costs. Compared with content supported by explicit numerical values, diagnostic labels, or direct imaging evidence, content that requires integration of dispersed information across the medical record and subsequent translation into communication needs may be more susceptible to cross-run variation. Safety-related outputs were also not completely stable. Pairwise agreement in case-level safety-event classification ranged from 87.5 to 95.8%, suggesting that unsupported cost inferences, misleading reassurance, or inappropriate role-boundary violations could appear in only one of the repeated runs. Therefore, the absence of a safety event in a single run does not establish stable safety performance for that case, and repeated generation cannot replace item-by-item human review of the actual output. The repeated-generation analysis was limited to 24 non-randomly selected cases. Case selection was not based on model coverage or safety outcomes, and no inferential statistical testing was performed. These findings should therefore be interpreted as exploratory evidence of substantive output variability rather than as a definitive estimate of the model's overall repeatability. Future studies should report both aggregate performance and direct item-level agreement and should further evaluate whether structured prompting, controllable generation parameters, and formal human-review procedures can reduce clinically meaningful output variability.

### 4.6 Limitations

This study has several limitations. First, only 80 cases from two centers were included, and the sample size was relatively limited. In addition, medical record documentation practices, regional clinical workflows, and payment environments may vary. Further validation is therefore needed in larger samples, multicenter settings, and different health care payment contexts. Second, the study population was limited to patients undergoing primary unilateral TKA. More complex scenarios, such as revision TKA, infection, bone tumors, complex post-traumatic arthritis, or combined major surgeries, were not included. These cases may differ from routine primary TKA in terms of surgical planning, material preparation, hospitalization pathways, complication risks, and cost communication needs. Therefore, the findings of this study cannot be directly generalized to all patients undergoing knee arthroplasty. Third, this study used an expert-referenced offline evaluation design. The POCC framework and case-level CL-MNCIs primarily reflected the professional judgments of joint arthroplasty surgeons, nursing specialists, and hospital cost-management personnel. The expert panel did not include patient representatives or frontline health insurance and billing personnel. In addition, the CL-MNCIs were established through joint expert review and consensus discussion rather than through independent generation followed by agreement assessment; therefore, inter-rater agreement was not calculated during development of the reference standard. Fourth, only a single model displayed in the publicly accessible ChatGPT interface during the study period was evaluated. The underlying model snapshot and generation parameters, including temperature, top-p, and random seed, could not be fixed by the researchers. Model updates and changes in platform configuration may therefore affect exact reproducibility. The repeated-generation analysis was also limited to 24 non-randomly selected cases and was intended primarily to explore variation in specific outputs; it should not be interpreted as a population-level statistical estimate of model stability. Finally, the CL-MNCI coverage rate and the proportion of case-relevant, record-supported generated content reflected coverage of necessary content and broad record support, respectively. Neither measure is equivalent to the overall quality or clinical effectiveness of the generated checklist. The study did not evaluate checklist usability, clinician editing burden, patient understanding, or communication outcomes in real clinician–patient interactions. The findings should therefore be interpreted as the LLM's offline performance under prespecified conditions. Future multicenter prospective studies should include clinicians, cost-management personnel, and patients and should evaluate real-world workflow integration and patient-related outcomes.

## 5 Conclusion

Using real-world preoperative TKA records from two hospitals and an expert-referenced offline evaluation design, this study evaluated the performance of ChatGPT-5.5 as a clinician-facing aid for preoperative cost-communication preparation. The model identified most clinical cues relevant to cost communication and covered most expert-defined case-level minimum necessary communication items. However, identification performance varied across labels, and omissions of necessary content, redundant or insufficiently supported generated content, and a small number of safety events remained. Repeated-generation analyses further showed that, although aggregate performance measures were similar across runs, specific label classifications, CL-MNCI coverage, and safety-event classifications were not completely stable.

These findings support the potential utility of LLMs as clinician-facing aids for preoperative cost-communication preparation in TKA. With item-by-item clinician review and multidisciplinary confirmation of specific treatment and payment information, such tools may improve the systematic organization and completeness of communication preparation. At present, however, LLMs should be used only as supervised communication-preparation aids and should not independently provide cost information to patients or replace professional cost-related judgment. Multicenter prospective studies are needed to further evaluate their clinical usability and patient-related value.

## Data Availability

The original contributions presented in the study are included in the article/Supplementary material, further inquiries can be directed to the corresponding authors.
